# Behind the Doors: 24 Insights Into Scientific Publishing

**DOI:** 10.32872/cpe.20861

**Published:** 2025-11-28

**Authors:** Cornelia Weise

**Affiliations:** 1 Department of Psychology, Clinical Psychology and Behavioral Health Technology, Friedrich-Alexander-Universität Erlangen-Nürnberg, Erlangen, Germany

How do you write a farewell editorial after having founded, built, and led a journal as Editor-in-Chief for seven years? And how do you do that at the end of the year, when many of us feel a little exhausted after another fully packed twelve months? This is the season when most of us are less eager to dive into the latest scientific breakthroughs and are more ready to simply count the days until things finally quiet down. The ever-buzzing inbox falls silent, pressing deadlines fade away, and even universities close for the holidays to save a little energy.

But perhaps that’s exactly the right mindset for this piece: counting days. So why not draw on that good old German tradition of the Advent calendar – opening one little door after another? Let’s open the doors and see what surprises (and lessons) they hold from my seven years as Editor-in-Chief. 

**#1 New Beginnings.** It all began in 2018, when we got in touch with Armin Günther, who was then the Managing Editor at the Leibniz Institute for Psychology (ZPID). We discussed the pros and cons of launching a new journal and quickly became convinced that this project would be a great and innovative way to increase the visibility of clinical psychology in Europe.

**#2 Open Access**. At the heart of CPE’s mission is the Diamond Open Access model, i.e., no fees for authors or readers. This model reflects our belief that scientific knowledge should circulate freely, independent of financial barriers or institutional privileges. My sincere thanks go to the Leibniz-Institute for Psychology for making this true open access possible.

**#3 Psychologists as Brand Designers**. Of all the tasks that came with launching the journal, designing our logo was one of the unexpected creative challenges. It turned out to be a lot of fun, and I’m still very happy with the result. Can you guess all the ideas hidden within it?

**#4 Team Spirit**. CPE has been a true team effort. Working with my co-editors and our editorial board made the journal what it is today. Sharing ideas, debating choices, and solving problems together was, and is, what kept it moving forward.

**#5 Innovation**. For a journal, innovation means staying curious and open to new ways of advancing scientific communication. At CPE, we welcome ideas that enhance both the journal’s visibility and the impact of clinical psychology. Our new Early Career Researcher Board is one step in that direction, helping shape CPE’s future and bring fresh ideas to life.

**#6 Editor’s St. Nicholas**. In an editor’s world, St. Nicholas wouldn’t fill shoes with sweets and oranges, but with submissions that follow the author guidelines, carefully revised manuscripts, or messages from reviewers who agree to review.

**#7 Cornerstones**. CPE rests on the steady support of its academic home, EACLIPT. Over the years, EACLIPT has provided stability and exchange, while giving us the freedom to shape the journal’s identity. We couldn’t have wished for a better foundation.

**#8 Copyediting Pro Tip**. When you submit your paper and receive detailed instructions on how to prepare your figures and tables, please make sure to follow them. Otherwise, the copyediting team might ask again (and again… and again) to fix the layout. We can’t help it. We’re a bit nerdy when it comes to the look of a final manuscript.

**#9 Responsibility**. An academic journal must actively protect and promote scientific freedom – especially when censorship or financial restrictions threaten research, and thus the progress needed to safeguard mental health. I’m deeply grateful that our association, EACLIPT, published a statement on the importance of academic freedom (Martin-Soelch et al., 2025). At CPE, we stand firmly by this commitment.

**#10 Student Assistants Do the Trick**. Behind every successful project is a great team. Over the years, several student assistants have supported CPE from submission to publication behind the scenes. Thank you, Juliane Haas, Ania Hoffmann Salán, Hannah Sandner, and Annkatrin Simon, for being such an essential part of the team.

**#11 Transparency**. For CPE, open science is not just a policy, it’s a commitment to trust. From the very beginning, we’ve taken transparent and reproducible research seriously. By implementing the Transparency and Openness Promotion (TOP) Guidelines (Center for Open Science, 2025), CPE actively contributes to making empirical research more transparent, credible, and verifiable.

**#12 Recommend Reviewers**. The better your reviewer suggestions, the sooner you’re likely to hear from us. And just to clarify: we won’t contact the colleague with whom you’ve co-authored 200 papers, the person in the next office, or anyone we can’t find in a five-minute web search.

**#13 Recommend Further Reviewers**. Yes, three potential reviewers are great, but an even longer list makes us truly happy. Keep in mind that it can take up to 15 invitations to secure just two reviewers who agree.

**#14 Backbone**. Peer review is the backbone of scientific self-regulation. Imperfect as it is, it ensures that ideas are challenged, refined, and strengthened through the scrutiny of one’s peers. I greatly appreciate all reviewers who shared their time and expertise.

**#15 Supplementary Material**. Once upon a time, in the 1990s, “Available upon request” was acceptable. Today, in 2025, transparency demands more. Upload your supplementary materials, and we’ll gladly assist via PsychArchives.

**#16 Core of Clinical Psychology**. CPE strives to highlight new developments that shape clinical psychology and its practice. Our special issue on mental health innovations in the ICD-11 illustrates this mission (Maercker, 2022). As a European journal, we aim to connect global advances with the realities of clinical work and training across Europe.

**#17 Curiosities**. Work can wait – curiosity can’t! For a quick mental break, take a look at last year’s Season’s Editorial on visualizations of sex and gender on toilet doors (Rosmalen et al., 2024). Who knows – perhaps it might inspire you to explore some unanswered questions of your own.

**#18 Impact**. One of the first topics we discussed with our publisher was how to achieve an impact factor as quickly as possible. Even though it took some time, we are grateful and proud that CPE is now listed in the Web of Science and has received its first – and already impressive – impact factor in 2025. CPE will continue to strive for excellence and to further strengthen this achievement.

**#19 Revise and Resubmit**. A second or third round of revision is rarely pleasant for authors. As editors, we share those sighs. It is challenging for us, too, to ask for more changes. Although the process can be time-consuming and frustrating, the outcome almost always speaks for itself: the papers become stronger. So, keep going; it's worth it.

**#20 The Invisible Architecture**. No journal thrives without the steady, precise, and patient work of its publisher. People who quietly build up the system, organize workflows, answer support questions, and step in at the last minute to keep everything running. For CPE, the Leibniz Institute for Psychology (ZPID) has been this foundation. Without it, many of our ideas would never have left the drawing board.

**#21 Christmas Reading**. For those celebrating Christmas, I’d like to highlight our 2022 editorial on how singing under the Christmas tree can positively impact mental health (Kanske & Rief, 2022). It’s well worth revisiting at this time of year.

**#22 Special Issues**. We’ve published three special issues so far, each capturing a snapshot of a particular research area. My personal favourite is the one on cultural adaptation of psychological interventions. It brings together various approaches to culturally sensitive psychotherapy and provides clear guidelines for reporting cultural adaptations in clinical trials, making it a key resource for transcultural research worldwide (Heim & Weise, 2021).

**#23 The Joy of Discovery**. Amid the long list of editorial to-dos, there is one moment that stands out: when you read a submission and suddenly pause. A novel idea, an unexpected dataset, or a brilliant line of reasoning emerges. And for a moment, you remember why you entered academia in the first place: the quiet thrill of discovering something new.

**#24 Farewell**. Over these years, I have learned a great deal, not only about academic publishing, impact, indexing, and copyediting, but also about responsibility, tackling challenges, and working under a very different kind of time pressure. Along the way, I’ve met many fascinating people and am very grateful for the projects, collaborations, and initiatives that grew out of these encounters. I am especially thankful for the support of my wonderful co-editors, Winfried Rief and Nadine-Messerli-Bürgy, and our colleagues at ZPID, particularly Judith Tinnes, Gerrit Fröhlich, and Armin Günther. Building this journal together was more often joy than work, and even when things didn’t go as planned, our collaboration always stayed constructive. My gratitude also goes to my colleagues and friends who suggested topics, authors, or reviewers (and occasionally stepped in themselves). And to my own research team – thank you for your patience whenever a new journal issue took precedence over our own papers.

CPE will always remain close to my heart. I’m truly grateful to have served as Editor-in-Chief for so many years, and I wish the continuing and new editors a confident, inspired hand and every success in using CPE to strengthen the visibility of clinical psychology in all its relevance, excellence, and diversity.
